# HIV-1 Envelope Glycoprotein Variable Loops Are Indispensable for Envelope Structural Integrity and Virus Entry

**DOI:** 10.1371/journal.pone.0069789

**Published:** 2013-08-01

**Authors:** Tingting Yuan, Jingjing Li, Mei-Yun Zhang

**Affiliations:** AIDS Institute, Department of Microbiology, Li Ka Shing Faculty of Medicine, The University of Hong Kong, Hong Kong, China; Institut Pasteur, France

## Abstract

HIV-1 envelope (Env) glycoprotein is a trimer of heterodimer of gp120 and gp41, and derives from a trimeric glycoprotein precursor, gp160. Gp120 contains five conserved regions that are interspersed with 5 variable loop regions (V1–V5). Env variations in variable loop length and amino acid composition may associate with virus pathogenesis, virus sensitivity to neutralizing antibodies (nAbs) and disease progression. To investigate the role of each variable loop in Env function, we generated a panel of JRFL gp160 loop deletion mutants and examined the effects of each loop deletion on Env expression, Env cell surface display and Env-mediated virus entry into permissive cells. We found that deletion of V1 and V2 (ΔV1V2), or loop D (ΔlpD) abolished virus entry, the same effect as deletion of V3 (ΔV3), while deletion of V3 crown (ΔV3C) significantly enhanced virus assembly and entry. We further found that deletion of V4 (ΔV4) or V5 (ΔV5), or replacement of V4 or V5 with flexible linkers of the same lengths knocked out the receptor and coreceptor binding sites in gp120, but significantly enhanced the exposure of the N-trimer structure and the membrane proximal external region (MPER) in gp41. Although deletion of V4 or V5 did not affect Env expression, they negatively affected Env cell surface display, leading to the failure in virus assembly and subsequent entry. Taken together, we found that Env variable loops were indispensable for Env structural integrity and virus entry. Our findings may have implications for development of HIV-1 vaccine immunogens and therapeutics.

## Introduction

HIV-1 envelope glycoprotein (Env) is a trimer of heterodimer of the mature surface glycoprotein gp120 and the transmembrane glycoprotein gp41. Env trimer mediates virus entry into permissive cells upon binding to the receptor (CD4) and coreceptor (CCR5 or CXCR4). Env gp120 contains five variable loops (V1–5) that are interspersed with five sequence conserved regions (C1–5). Despite sequence and length variations, Env variable loops are important determinants or indicators for coreceptor tropism, virus sensitivity to neutralization by antibodies, virus pathogenesis, and disease progression [1], [2], [3], [4], [5], [6], [7], [8], [9]. It is well studied that V3 is the primary determinant of viral coreceptor usage although mutations outside the V3 have impact on coreceptor tropism of subtype B viruses [3]. V1V2 region may influence coreceptor binding and participate in shielding of neutralization-sensitive regions of the Env. V1V2 length and potential N-linked glycosylation sites (PNGS) were found to increase significantly through chronic infection before declining in late-stage infection [1], and an increase in the V1V2 length and/or the number of PNGS in the V1V2 region directly contributed to viral resistance to HIV-specific neutralizing antibodies (nAbs) [4], [10]. Important structural motifs formed by the C3 and V4 regions and the epitopes within the motifs were also found to be major targets of the early autologous neutralizing response in HIV-1 subtype C infection [7]. Extensive intra-patient V4 variability in length and number of PNGS has also been observed in clade B, G, and CRF02 isolates during early infection [2]. In addition, some variable loops constitute neutralizing determinants recognized by several known broadly neutralizing HIV-1 human monoclonal antibodies (bnmAbs). For example, bnmAb VRC01 binds to an epitope formed by the CD4 binding site (CD4bs), V5 and loop D (lpD) [11], and bnmAbs PG9/16, CH04 and PGT145 are V1/V2-directed antibodies [12], while a short β-strand segment of the V3 loop is involved in binding of PGT127 and 128 to Env trimer [13]. These observations suggest potentially important roles of variable loops in Env-mediated virus entry and virus pathogenesis. However, how changes in these loops affect Env function are not well studied.

In this study, we generated a series of loop deletion or replacement mutants of JRFL gp160, and investigated the effects of each loop deletion or replacement on Env expression, Env cell surface display, and virus assembly and subsequent virus entry into permissive cells. In addition to the five variable loops, we also investigated the importance of two other loops, the CD4 binding loop (CD4bl) and lpD, for Env-mediated viral function. We further investigated if deletion of Env cytoplasmic tail (CT) can rescue the defects in Env expression and function caused by variable loop deletions.

## Materials and Methods

### Cells, Plasmids, Medium, Antibiotics, and Antibodies

93 T cells were purchased from ATCC. TZM-bl cell line was obtained from the NIH AIDS Research and Reference Reagent Program (ARRRP). The pSVIII-JRFL gp160 wild type (WT) and pcTAT plasmids were kindly provided by Yuxing Li, Richard Wyatt and Joseph Sodroski [14]. DMEM medium, fetal bovine serum (FBS) and penicillin-streptomycin (pen-strep) were purchased from Gibco. Purified gp120-specific polyclonal Abs D7324 were purchased from Aalto BioReagents. IgG1s b12 and 2G12 were obtained from ARRRP. IgG1 VRC01 was kindly provided by Xueling Wu and John Mascola. The rest of HIV-1 mAbs were produced in our laboratory by transient transfection of 293F cells (Invitrogen) followed by Protein A (GE Healthcare) affinity purification.

### Construction of Loop Deletion Mutants

Full-length JRFL gp160 gene in pSVIII was used as a template for generation of various loop deletion or replacement mutants, including single loop deletion of V2 (ΔV2), V3 (ΔV3), V4 (ΔV4), V5 (ΔV5), loop D (ΔlpD), CD4 binding loop (ΔCD4bl), and deletion of V2 crown (ΔV2C) and V3 crown (ΔV3C), and double loop deletion of V1 and V2 (ΔV1V2), and loop D and V5 (ΔlpDΔV5), and replacement of V4 (ΔV4fl) and V5 (ΔV5fl) with flexible linkers of the same lengths (Table 1). A panel of primers were designed and synthesized for amplification of different fragments of JRFL gp160 gene and for replacement of each loop with either a short flexible linker or a flexible linker of the same length as the original loop (in the case of V4 and V5) (Table 1 and 2). Primer pSV3for was paired with each anti-sense primer to amplify the upstream fragments of the Env, and primer pSV3rev was paired with each sense primer for amplification of the corresponding downstream fragments using the following PCR program: an initial denaturation at 94°C for 5 min, followed by 10 cycles of 95°C for 20 s, 50°C for 30 s and 72°C for 90 s, and 20 cycles of 95°C for 20 s, 55°C for 30 s and 72°C for 90 s, and a final extension at 72°C for 10 min. The upstream and the corresponding downstream fragments for each mutant were assembled by splice overlap extension (SOE) as follow: an initial denaturation at 94°C for 5 min, followed by 5 cycles of 95°C for 20 s, 55°C for 30 s and 72°C for 90 s. The assembled full-length gp160 loop deletion mutant genes were then amplified by PCR using pSV3for and pSV3rev as a pair of primers and the following PCR program: 20 cycles of 95°C for 20 s, 55°C for 30 s and 72°C for 3 min, and a final extension at 72°C for 10 min. All PCR products were gel-purified using QIAquick gel extraction kit (Qiagen), digested with KpnI and BamHI, and ligated to pSV3 plasmid digested with the same restriction enzymes. Ligation products were used to transform TG1 electroporation competent cells. Each loop deletion or replacement mutant was confirmed by DNA sequencing.

**Table 1:** JRFL gp160 loop deletion or replacement mutants.

JRFL Env loops	Original loop sequence	Replacing linker sequence and designated name of the construct
**V1V2**	VNATNTTNDSEGTMERGEIKNCSFNITTSIRDEVQKEYALFYKLDVVPIDNNNTSYRL	GSGSG (ΔV1V2)
**V2**	NITTSIRDEVQKEYALFYKLDVVPIDNNNTSYRL	GSGSG (ΔV2)
**V2 crown**	FYKLD	AAAAA (ΔV2C)
**loop D**	NFTNNAKT	AAAA (ΔlpD)
**V3**	NTRKSIHIGPGRAFYTTGEIIG	GSGSG (ΔV3)
**V3 crown**	GPGR	AAAA (ΔV3C)
**CD4bl**	SSGGDPEIVMH	GSGSG (ΔCD4bl)
**V4**	NSTQLFNSTWNNNTEGSNNTEGNTITLP	GSGSG (ΔV4); or GGGGSGGGGSGGGGSGSGSG (ΔV4fl)
**V5**	INENGTEIFR	GSGSG (ΔV5); or GGGGSGSGSG (ΔV5fl)

The amino acid (AA) sequences of the original loops and flexible linkers for replacement are shown. V2 and V3 crown sequences are underlined. CD4bl: CD4 binding loop. ΔV4fl and ΔV5fl: V4 and V5 loops replaced with flexible linkers of the same lengths. Designated names of resultant constructs are indicted in parentheses.

**Table 2:** Primers used to construct JRFL gp160 loop deletion mutants.

Primer Name	Primer Sequence (5′ to 3′)	Use
pSV3for	ACCATGCTCCTTGGGATGTTGATG	annealing to plasmid pSVIII
pSV3rev	TCTCAAGCGGTGGTAGCTGAAGAG	annealing to plasmid pSVIII
delLpDF	GACGCTGCAGCAGCTATAATAGTACAGCTGAAAGAATC	generating ΔlpD
delLpDR	AGCTGCTGCAGCGTCAGATCTAATTACTACCTC	
Delv1v2F	GGTAGCGGATCAGGTATAAGTTGTGACACCTCAGTC	generating ΔV1V2
Delv1v2R	ACCTGATCCGCTACCATCCTTGCAATTTAAAGTAAC	
V2crownF	GCTGCTGCAGCTGCTGTAGTACCAATAGATAATAATAATACC	generating ΔV2C
V2crownR	AGCAGCTGCAGCAGCAAGAGCATATTCTTTCTGCAC	
delfullV2F	GGTTCAGGATCTGGCATAAGTTGTGACACCTCAGTC	generating ΔV2
delfullV2R	GCCAGATCCTGAACCGAAAGAGCAGTTTTTTATTTCTC	
V3crownF	TAGCAGCAGCTGCAGCATTTTATACTACAGGAG	generating ΔV3C
V3crownR	CTGCAGCTGCTGCTATATGTATACTTTTTCTTG	
Delfullv3F	GGTTCTGGATCAGGTGATATAAGACAAGCACATTGTAAC	generating ΔV3
Delfullv3R	ACCTGATCCAGAACCGTTGTTGGGTCTTGTACAATTAATTTC	
V4delFnew	GGCTCTGGTTCTGGGTGCAGAATAAAACAAATTATAAACATG	generating ΔV4 with a short linker
V4delRnew	CCCAGAACCAGAGCCACAGTAGAAAAATTCTCCTCCAC	
V5delFnew	GGTAGCGGATCAGGCCCTGGAGGAGGAGATATGAG	generating ΔV5 with a short linker
V5delRnew	GCCTGATCCGCTACCACCACCATCTCTTGTTAATAG	
DelCD4blF	GGTTCTGGATCAGGTAGTTTTAATTGTGGAGGAGAATTTTTC	generating ΔCD4bl
DelCD4blR	ACCTGATCCAGAACCGTGATTAAAGACTATTGTTTTATTCTC	
V4FFL	GGTGGAGGCGGTTCAGGCGGAGGTGGCTCTGGCGGTGGCGGATCA GGCTCTGGTTCTGGG	generating ΔV4 with a linker of the same length
V4RFL	CCGCCTGAACCGCCTCCACCACAGTAGAAAAATTCTCCTCCAC	
V5FFL	GGTGGAGGCGGTTCAGGTAGCGGATCAGGCCCTGGAGG	generating ΔV5 with a linker of the same length
V5RFL	CTACCTGAACCGCCTCCACCACCACCATCTCTTGTTAATAG	
JRFLCTfor	CCTGGATGGAGTGGGAAAGAG	generating ΔCT
JRFLCTrev	CTCTTTCCCACTCCATCCAGG	

### Transient Transfection of 293 T Cells and Flow Cytometry

The day before transfection, 293 T cells were seeded in DMEM growth medium (DMEM containing 10% FBS and 1% pen-strep). 70–80% confluent 293 T cells were co-transfected with pSVIII expression plasmid containing JRFL wild type (WT) gene or its loop deletion or replacement mutants, and pcTAT using PEI as a transfection reagent in DMEM medium containing 10% FBS. 4–6 h post transfection, the medium was changed to DMEM growth medium. 48 h post transfection, 293 T cells were harvested, washed with FACS buffer (PBS+5% FBS) and stained with IgG1 2G12, or VRC01, or b12 as a primary antibody at 4°C for 1 h. Following washing three times with FACS buffer, cells were incubated with phycoerythrin (PE) conjugated to anti-human Fc (Sigma) (1∶200) at 4°C for 45 min. Cells were then washed again three times with FACS buffer and analyzed on a BD flow cytometer. The results were analyzed by FlowJo software.

### Generation of Env-pseudotyped Viruses

Env-pseudotyped viruses were assembled by co-transfection of 293 T cells with Env plasmid containing JRFL Env WT gene or its loop deletion or replacement mutants with or without the CT, and HIV-1 backbone plasmid pNL4-3 containing a luciferase reporter gene and HIV-1 structural genes. 48 h post transfection, the culture supernatants containing assembled pseudovirus were harvested and stored at −80°C, or directly used in virus titration by capture ELISA and subsequent virus entry assay.

### Pseudovirus Entry Assay

Crude preparation of pseudovirus in culture supernatant was 3-fold serially diluted in DMEM growth medium and 100 µl of each dilution in triplicate were placed in 96-well flat-bottom cell culture plates. 100 µl of TZM-bl cell suspension in DMEM growth medium containing DEAE-Dextran (25 µg/ml) were dispensed to the wells to a final cell density of 10,000 cells per well and a final concentration of DEAE-Dextran of 10 µg/ml. The plates were then incubated at 37°C with 5% CO2 for 48 h. Cells were washed with PBS once and lysed with 60 µl per well lysis buffer by incubating at RT for 30 min followed by addition of 25 µl of luciferin (Promega). Luminescence readings (RU) were determined by PE Victor3 luminometer. Relative pseudovirus entered into the cells was calculated as follow: (RU_sample_-RU_blank_)/(RU_WT_-RU_blank_)×100.

### Capture ELISA

250 ng per well of D7324 in PBS were coated on high-binding 96-well half-well microplates by incubating the plates at 4°C overnight. Following blocking the wells with 2.5% skim milk in PBS (MPBS), 3-fold serially diluted whole cell lysate or pseudovirus-containing culture supernatant in lysis buffer (2% Triton X-100 from Sigma in PBS) were added to the ELISA plates. Captured JRFL gp160s were detected by using IgG1 2G12 as a primary antibody and HRP conjugated to anti-human F(ab’)2 (1∶ 2,500) as a secondary antibody, and ABTS (Roche) as substrate. The optical density at 405 nm (OD405nm) was determined after color development at RT for 20 min. A similar capture ELISA set-up was used to determine structural integrity of loop deleted or replaced JRFL gp160 by measuring the binding of a panel of HIV-1-specific mAbs to each loop deleted or replaced Env mutant and WT captured on microplates. HIV-1-specific mAbs include CD4bs mAbs VRC01 and b12, CD4-induced (CD4i) mAbs X5 and 17b, and gp41-specific mAbs m47 (N-trimer-specific, unpublished), 2F5 and 4E10. Glycan-specific mAb 2G12 was included in the assay to measure each loop deleted or replaced Env mutant expressed in 293 T cells. A standard curve used for data calibration was generated using recombinant JRFLgp120 as antigen and 2G12 as a primary antibody in the same capture ELISA.

### Immunohistochemistry (IHC) Assay

48 h post transfection, 293 T cells were fixed using 4% paraformaldehyde fixative solution by incubation at RT for 20 min. If necessary, cells were permeabilized using 0.2% Triton X-100 by incubation at RT for 30 min. Cells were then incubated with IgG1 2G12 in 2% MPBS at 37°C for 1 h, washed two times with PBS, and incubated with FITC conjugated to anti-human Fc (Jackson ImmunoResearch) at 37°C for 1 h. Following washing two times with PBS, cells were permeabilized using 0.2% Triton X-100 by incubation at RT for 30 min, and incubated with Hoechst 33258 (Invitrogen) at RT for 2 min to stain the nucleus.

## Results

### Effects of Variable Loop Deletions on Env Cell Surface Display

**Figure 1:**
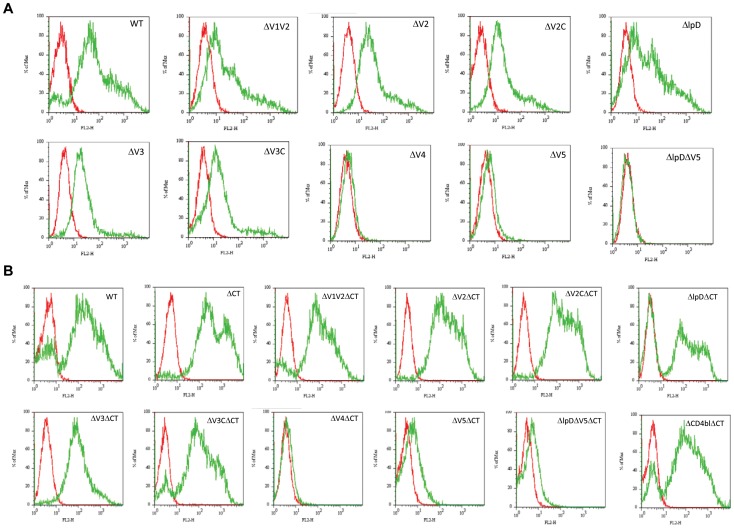
Effects of various loop deletions on Env cell surface display. A: Flow cytometry of 293T cells co-transfected with various loop deletion Env plasmids and pcTAT; B: Flow cytometry of 293 T cells co-transfected with various loop deletions and CT deletion plasmids and pcTAT. Cells were incubated with HIV-1 mAb 2G12 and bound 2G12 measured by FITC-anti-human IgG, F(ab’)2.

We co-transfected 293T cells with pSVIII encoding JRFL gp160 wild type (WT) or loop deletion mutants, and pcTAT encoding HIV-1 Tat. 48 h post transfection, we did flow cytometry to analyze cell surface display of Env gp160 WT and the mutants using mAb 2G12 as a primary antibody and PE-anti-human IgG, F(ab’)2 as a secondary antibody. We found that ΔV4, ΔV5, and ΔlpDΔV5 almost abolished Env cell surface display (Fig. 1A, Table 3). Other loop deletion mutants also decreased their cell surface display compared to the WT, including ΔV2, ΔV2C, ΔV3, ΔV3C, ΔlpD, and ΔV1V2 (Fig. 1A, Table 3).

**Table 3:** Effects of loop deletions with or without the CT deletion on JRFL gp160 cell surface display, virus assembly, and subsequent virus entry.

ENV variants (JRFL)	Mean value in flowcytometry	Relative Env cellsurface display (%)	Relative pseudovirus assembled (%)	Relative pseudovirus entry (%)
WT	312	100	100	100
JRFLΔV1V2	178	57	43	0
JRFLΔV2	148	48	442	31
JRFLΔV2C	159	51	258	35
JRFLΔlpD	87	28	137	0
JRFLΔV3	118	38	313	0
JRFLΔV3C	101	32	1,828	343
JRFLΔV4	9	3	1	NT
JRFLΔV5	9	3	6	NT
JRFLΔCT	457	147	2,259	6,375
JRFLΔV1V2ΔCT	255	82	55	0
JRFLΔV2ΔCT	217	70	1,733	91
JRFLΔV2CΔCT	437	140	1,095	253
JRFLΔlpDΔCT	428	137	612	0
JRFLΔV3ΔCT	236	76	1,822	0
JRFLΔV3CΔCT	320	103	5,669	1,195
JRFLΔV4ΔCT	6	2	1	NT
JRFLΔV5ΔCT	9	3	2	NT
JRFLΔlpDV5ΔCT	6	2	4	NT
JRFLΔCD4blΔCT	322	103	5,106	0

**Figure 2:**
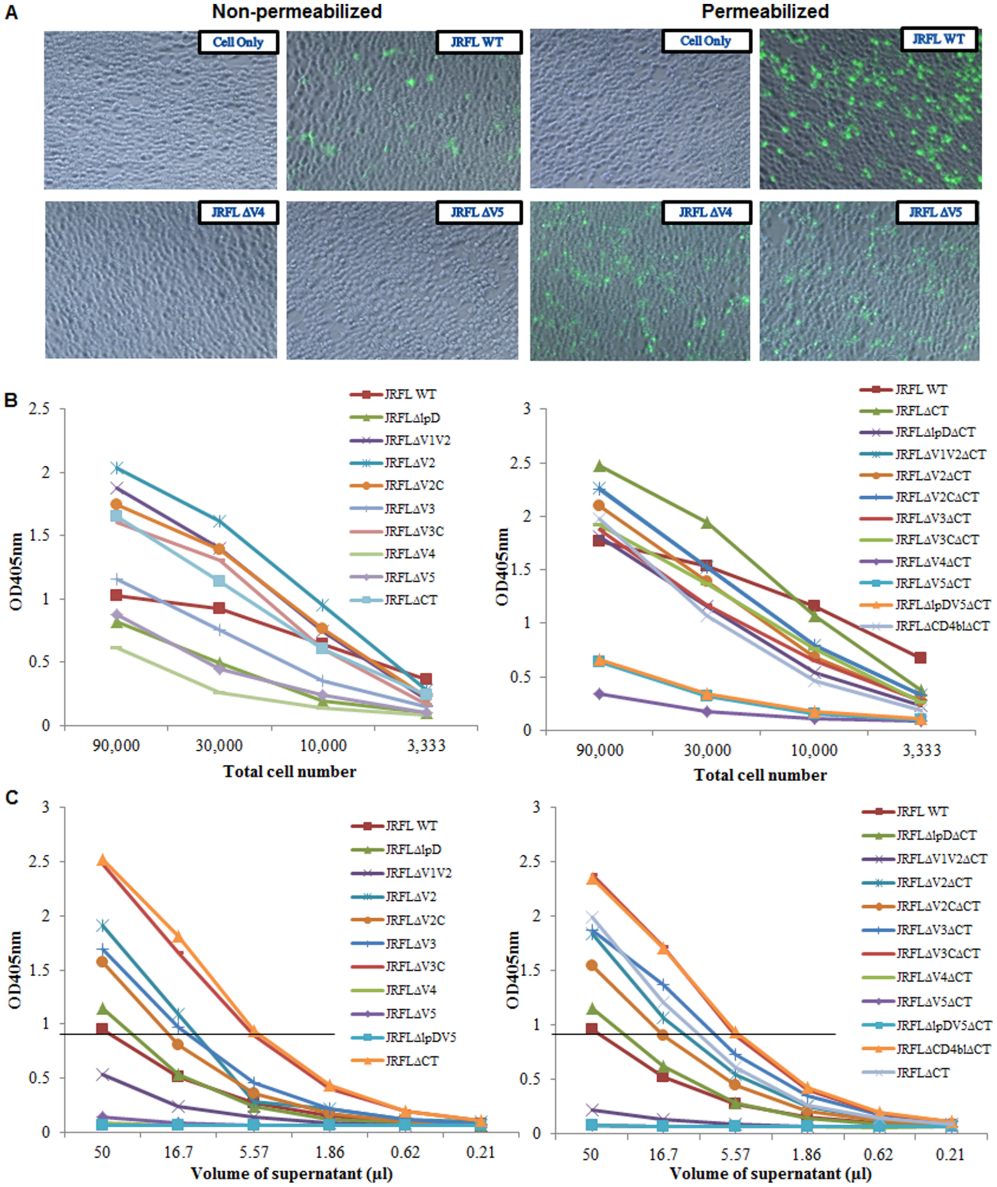
Effects of various loop deletions on total Env expression in 293T cells, Env binding to mAb 2G12, and pseudovirus assembly. A: Immunostaining of 293T cells co-transfected with ΔV4 or ΔV5 Env plasmid and pcTAT with or without permeabilization prior to staining with mAb 2G12; B: Binding of Env loop deletion mutant proteins in the whole cell lysates to 2G12 by capture ELISA; C: Titration of pseudovirus in the culture supernatants by capture ELISA. The volume of each supernatant that contains the same amount of pseudovirus is indicated with a flat line when OD405nm = 0.95.

Cell surface displayed Env proteins were measured by flow cytometry. Mean values are shown and cell surface displayed Env proteins relative to the WT are calculated as a percentage. The amount of assembled pseudovirus in culture supernatant was measured by capture ELISA. Three-fold serially diluted culture supernatants with a starting volume of 50 µl were tested in luciferase assay. Relative pseudovirus entry was defined as a percentage of luminescence reading (response units, RU) of each mutant virus relative to the WT when same amount of assembled pseudovirus was used. In this case, the same amount of pseudovirus gave OD405nm of 0.95 in the capture ELISA (Fig. 2C). NT: not tested.

To determine whether CT deletion can rescue the defects in cell surface display of Env loop deletion mutants, we deleted the CT in JRFL gp160 WT and each loop deletion mutant, and generated another panel of loop deletion mutants in combination with CT deletion (ΔCT) (Table 3). A CD4 binding loop (ΔCD4bl) deletion mutant in combination with ΔCT (ΔCD4blΔCT) was included in this panel of mutants and used as a control in the subsequent pseudovirus assembly and entry assay. We found that ΔCT alone significantly enhanced Env expression and cell surface display (Fig. 1B), which is consistent with the result previously reported by others. But deletion of CT did not rescue the defects of ΔV4 and ΔV5 Envs in cell surface display (Fig. 1B, Table 3). The other loop deletion mutants in combination with ΔCT had increased levels of cell surface display compared to the corresponding loop deletion alone mutants. The rescue was significant for ΔlpD mutant.

To determine whether decreased cell surface display of ΔV4 and ΔV5 Envs was caused by decreased transfection efficiency or decreased Env expression, we measured the transfection efficiency by IHC assay and determined the total ΔV4 and ΔV5 Env expression in the whole cell lysates of transfected 293T cells by capture ELISA using 2G12 as primary antibody. The percentages of positive cells transfected with ΔV4 and ΔV5 Env plasmids, as well as the rest of loop deletion mutant plasmids showed no significant difference compared to the WT (Fig. 2A, and data not shown), suggesting that loop deletion mutant plasmids can transfect 293T cells as efficient as the WT plasmid. Capture ELISA result showed that ΔV4 and ΔV5 Envs were well expressed in the cells albeit to a decreased optical density (OD) at 405nm (Fig. 2B), which may attribute to the loss of PNGS in the V4 and V5, leading to decreased binding to 2G12. The rest of loop deletion mutants expressed well in 293T cells and bound well to 2G12 (Fig. 2B).

### Effects of Variable Loop Deletions on Virus Assembly and Entry into Permissive Cells

The defects of ΔV4 and ΔV5 Envs in cell surface display led to the failure in pseudovirus assembly and subsequent virus entry (Fig. 2C, Table 3). Assembled pseudovirus was undetectable in the culture supernatant of 293T cells co-transfected with pSVIII-ΔV4, or -ΔV5, or -ΔlpDV5, and pcTAT and pNL4-3 backbone plasmids. Interestingly, we found that ΔV1V2 also affected virus assembly (Fig. 2C), and abolished virus entry (Table 3). More interestingly, ΔlpD did not affect virus assembly (Fig. 2C), but the assembled pseudovirus was not able to enter the permissive cells (Table 3). Both ΔV2 and ΔV2C enhanced virus assembly, but negatively affected virus entry. As expected, ΔV3 abolished virus entry although the amount of assembled pseudovirus increased. To our surprise, ΔV3C led to almost 20-fold increase in virus assembly and 3-fold increase in virus entry compared to the WT (Table 3). Deletion of CT in the WT Env significantly enhanced pseudovirus assembly and subsequent entry into the cells, which is consistent with the observation previously reported by others. But deletion of CT did not rescue ΔV1V2 and ΔlpD viruses to enter the cells although ΔlpDΔCT had 5-fold increase in pseudovirus assembly compared to ΔlpD alone (Table 3). Interestingly, we found that ΔCT significantly enhanced assembly of ΔV2, ΔV3, ΔV2C, and ΔV3C pseudoviruses (3-6-fold increases), and enhanced the entry of ΔV2C and ΔV3C pseudoviruses into the cells (Table 3). Combination of ΔCD4bl with ΔCT (ΔCD4blΔCT) also significantly enhanced pseudovirus assembly compared to the WT (50-fold increase) although the assembled pseudoviruses could not enter the cells, as expected (Table 3).

### Deletion of V4 or V5 did not Cause gp120 Shedding

To investigate the possibility that ΔV4 and ΔV5 may enhance gp120 shedding, resulting in lack of Env cell surface display, we did a capture ELISA to detect soluble gp120 that may be present in the culture supernatant of 293T cells co-transfected with recombinant pSVIII plasmid encoding JRFL gp160 WT, or ΔV4, or ΔV5, and pcTAT plasmid. IgG1 2G12 was used as a primary antibody in the capture ELISA. The result showed that gp120 was absent in the culture supernatant of 293T cells co-transfected with the pSVIII gp160 ΔV4 or ΔV5 mutant plasmid, and pcTAT, while gp120 was present in the culture supernatant of 293T cells co-transfected with Env WT plasmid and pcTAT (data not shown). This result indicates that undetectable ΔV4 and ΔV5 Envs on cell surface may be attributed to the lack of Env cell surface display, not to gp120 shedding.

### Effects of V4 and V5 Loop Deletions on Env Structural Integrity

**Figure 3:**
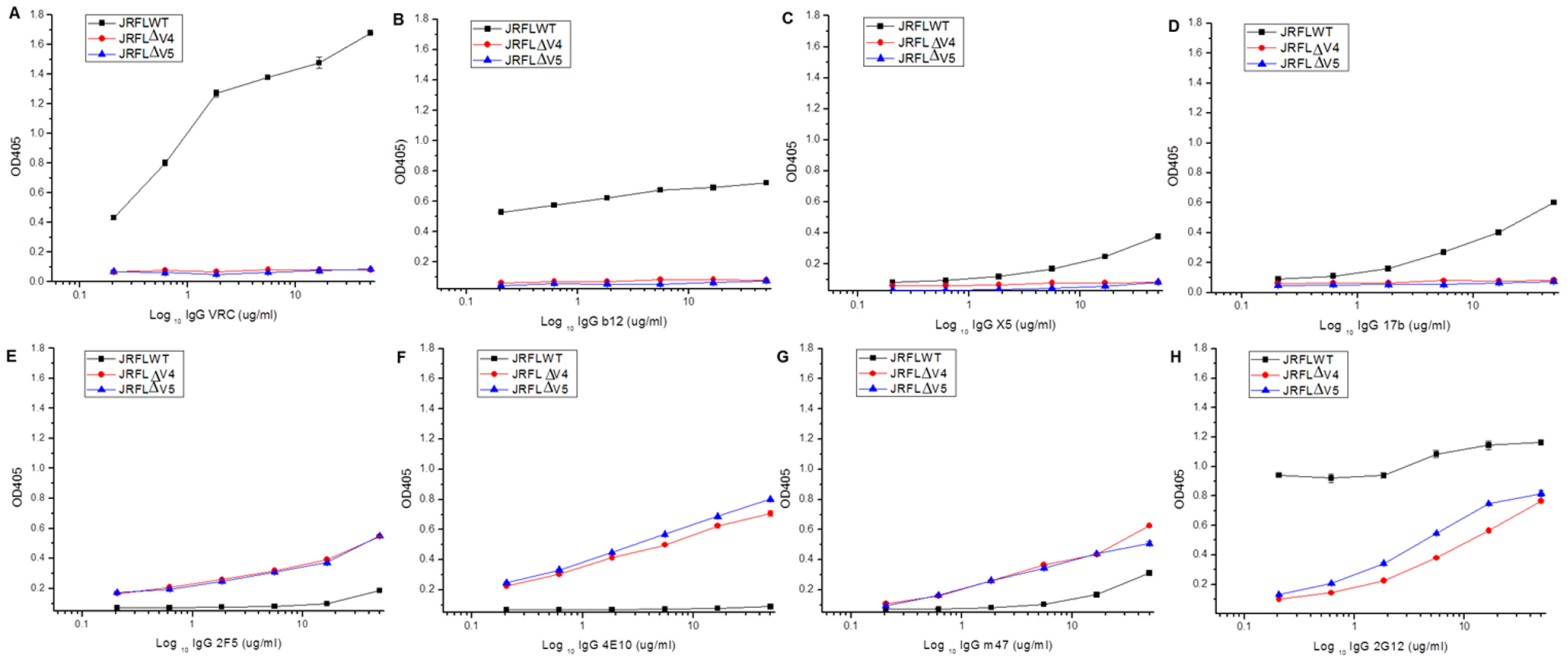
Effects of V4 and V5 loop deletions on Env structural integrity. 293T cells were co-transfected with recombinant pSVIII plasmid encoding JRFL gp160 WT, or ΔV4, or ΔV5 mutant and pcTAT plasmid. 48h post transfection, the transfected cells were collected and lysed with cell lysis buffer, and added to the ELISA plates coated with anti-HIV antibody D7324. Captured Env proteins were detected using gp120-specific CD4bs mAbs VRC01, b12, or CD4i mAbs X5, 17b, or glycan-specific mAb 2G12, or gp41-specific mAbs 2F5, 4E10, m47 as primary antibody and HRP-anti human IgG, F(ab’)_2_ as secondary antibody.

Since ΔV4 and ΔV5 Env proteins can be expressed in cells, but cannot be displayed on cell surface, we examined the possible conformational changes occurred to the Env proteins by measuring their bindings to various mAbs by ELISA. We found that ΔV4 and ΔV5 Env proteins lost the binding to CD4bs mAbs b12 and VRC01, and CD4i mAbs X5 and 17b, but had increased binding to gp41-specific mAbs m47 (N-trimer-specific)(unpublished), 2F5 and 4E10 (MPER-specific) (Fig. 3A-G). The result indicates that deletion of V4 or V5 may destroy Env structural integrity, resulting in loss of the receptor and coreceptor binding sites, and enhanced exposure of the N-trimer structure and the MPER. Binding of glycan-specific mAb 2G12 to ΔV4 and ΔV5 Env proteins decreased, to some extent, which may be attributed to the decreased number of PNGS in the Envs due to the deletion of V4 and V5 (Fig. 3H).

### Effects of V4 or V5 Replacement with a Flexible Linker of the Same Length on Env Expression and Function

**Figure 4:**
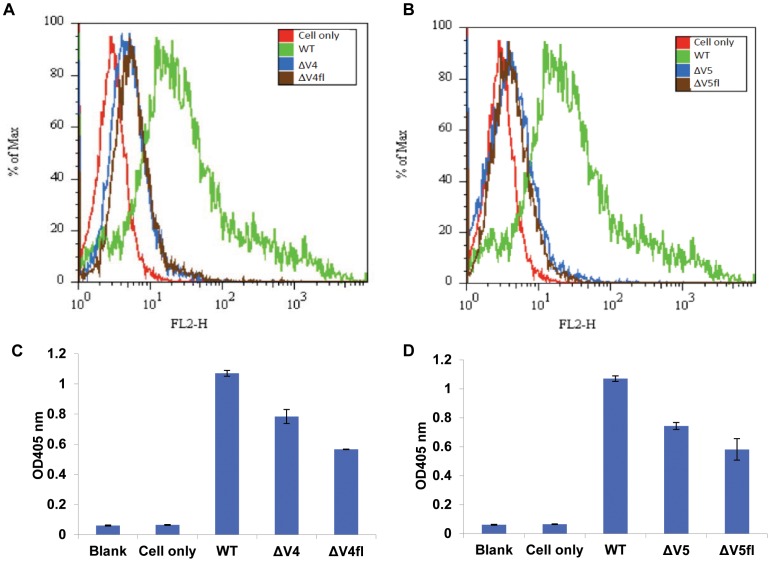
Expression of Env mutants with V4 and V5 replaced with flexible linkers of the same lengths as the original loops in 293T cells as measured by flow cytometry (A and B) and ELISA (C and D). 293T cells were co-transfected with recombinant pSVIII plasmid encoding JRFL gp160 WT, or Env mutants with V4 (A and C) or V5 (B and D) replaced with a flexible linker of the same length as the original loop, and pcTAT plasmid. 48h post transfection, the cells were detached and stained with 2G12 as primary antibody and PE-anti-human IgG, F(ab’)2 as secondary antibody (A and B), or cells were lysed with cell lysis buffer and the expressed Envs in the cell lysate captured by anti-HIV antibody D7324 (5 µg/ml) coated on high binding ELISA plates and bound Env proteins measured by using 2G12 as primary antibody and HRP-anti human IgG, F(ab’)2 as secondary antibody (C and D).

To investigate if V4 and V5 length, or amino acid composition, or both contribute to the importance of V4 and V5 for Env cell surface display and virus assembly, we generated two loop replacement mutants, designated ΔV4fl and ΔV5fl, in which V4 or V5 was replaced with a flexible linker of the same length as the original loop (Table 1). Flow cytometry and ELISA results showed that there were no significant differences between ΔV4 and ΔV4fl, or between ΔV5 and ΔV5fl in Env cell surface display (Fig. 4A and 4B), and in total Env protein expression in the cells (Fig. 4C and 4D), suggesting that amino acid composition of V4 and V5 may play a more important role than the loop length in preserving Env structural integrity and Env function.

## Discussion

Engineering Env is one of approaches for HIV-1 vaccine development. Understanding the importance of variable loops for Env-mediated viral functions may help develop Env-based vaccine immunogens and therapeutics against HIV-1 infection. Env CD4 binding loop and V3 are known to be involved in binding to the receptor and coreceptor during virus entry. In this study, we found that V1V2 and loop D were also critical for Env-mediated virus entry into permissive cells. Therefore, V1V2 and loop D could be key components of vaccine immunogens. Indeed, follow-up studies on the recent RV144 trial demonstrate the importance of V1V2 loop for inducing protective antibodies against HIV-1 infection [15]. Furthermore, V1V2 and loop D constitute partially the neutralizing epitopes of bnmAbs PG9/16 and VRC01, respectively. The critical roles of V1V2 and loop D in virus entry may also be explored for developing entry inhibitors targeting these regions. We did sequence alignment and found that six out of eight amino acids in loop D were highly conserved in all HIV-1 subtype viruses and the rest two relatively conserved (data not shown). Targeting conserved loop D may be an alternative to current approaches that target the CD4bs and/or glycans on Env trimer. We further found that V4 and V5 were indispensable for Env structural integrity, but deletion of V4 or V5 enhanced the exposure of the N-trimer structure and the MPER of Env. Several MPER-specific bnmAbs have been identified, including 2F5, 4E10, Z13e1 and 10e8 [16], [17], and many MPER-based peptides have been designed, but they failed to induce the same or similar bnAbs. Our ΔV4 and ΔV5 Env proteins may be tested in animals (rabbits or rhesus macaques) for inducing MPER-specific bnAbs. Engineered Env with enhanced exposure of the MPER may help induce MPER-specific nAbs in vivo.

Recently revealed cryo-EM structure of JRFL Env trimer suggested the involvement of V1V2 and V3 in gp120 trimer association [18]. Deletion of V1V2 may result in a decreased association of gp120 with Env trimer. Therefore, viral escape from nAbs by increased length and increased number of PNGS in V1V2 region during chronic infection may come at the cost of viral fitness. Loop D may be directly or indirectly involved in Env interaction with the receptor CD4, so deletion of loop D abolished virus entry. This notion is supported by the observation that amino acid substitutions in loop D cause viral escape from CD4bs bnmAb VRC01 [19]. Interestingly, we found that deletion of V2, or V2 crown, or V3, or V3 crown significantly enhanced pseudovirus assembly, especially when the CT was also deleted (Table 3), but the effects of these deletions on pseudovirus entry were different. Deletion of V2 or V2 crown negatively affected psudovirus entry, while deletion of V3 abolished pseudovirus entry. But to our surprise, we found that deletion of V3 crown significantly enhanced pseudovirus entry, and this enhancement was even more significant in the absence of CT. When the CT was deleted, deletion of V2 crown also enhanced psudovirus entry. Both V2 crown and V3 crown sequences were conserved, and V3 crown was very immunogenic and served as a target for developing V3-specific nAbs and inhibitory peptides [20]. It was postulated that V3 crown was critical for coreceptor binding based on the observation that V3 crown structurally mimicked the β2-β3 loop in the CXC and CC chemokines [21]. But our study indicated that V3 crown may not be involved in binding to the coreceptor (either CCR5 or CXCR4). This is supported by two observations. First, V3 crown was not involved in coreceptor selectivity [21]. Second, antibodies targeting V3 stem showed broad neutralizing activity [22].

Deletion of V4 or V5 did not lead to the failure in Env protein expression, but significantly affect Env cell surface display, as well as pseudovirus assembly and subsequent entry into the cells. According to the crystal structure of gp120 core containing V4 and V5, deletion of V4 essentially removed a conserved glycan at position 386 and half of the beta sheet-19, which may destroy the CD4 binding site, while deletion of V5 removed half of the beta sheet-24, which may in turn destabilize the neighboring CD4 binding loop. Nevertheless, expression of ΔV4 and ΔV5 Envs in the cells was not significantly affected. Failure in exporting ΔV4 and ΔV5 Envs to cell surface suggested that V4 and V5 may be involved in Env binding to host factors and/or matrix, which is required for transportation machinery to display Envs on cell surface. Loss of Env structural integrity caused by deletion of V4 or V5 may be associated with the loss of binding of Env to the host factors and/or matrix [23]. We found that replacement of V4 or V5 with a flexible linker of the same length did not alleviate the defects in Env cell surface display compared to the loop deletion mutants (replaced with significantly shorter linkers), indicating that V4 and V5 lengths may not be determining factors for virus sensitivity to nAbs and disease progression. Instead, increased numbers of PNGS in V4 and V5 along with the increased loop lengths may play more important roles in viral escape from nAbs and disease progression.

We tested the effect of ΔCT in combination with each loop deletion on Env protein expression, Env cell surface display, and pseudovirus assembly and subsequent entry. HIV-1 Env CT is unusually long (150 amino acids) and highly conserved. It affects Env cell surface display and subsequent virus assembly and entry [23], [24]. Interaction between matrix and CT is required for Env incorporation into membrane and virus infectivity [25]. In this study, we also observed that ΔCT alone led to increased Env expression and Env cell surface display, and significantly enhanced pseudovirus assembly and entry. But deletion of CT did not rescue the defects of ΔV4 and ΔV5 Envs in cell surface display, and in pseudovirus assembly and entry into the cells, suggesting that V4 and V5 deletion or replacement may be detrimental to Env structure and function.

### Conclusions

HIV-1 Env variable loops are indispensable for Env structural integrity and Env-mediated virus entry. Deletion of V1V2, or V3, or loop D abolished virus entry. Deletion of V4 or V5, or replacement of V4 or V5 with flexible linkers of the same lengths knocked out receptor and coreceptor binding sites in gp120, but enhanced the exposure of the N-trimer structure and the MPER in gp41. Deletion of V4 or V5 significantly affected Env cell surface display, leading to the failure in virus assembly and subsequent entry. Our findings suggest that, in addition to the CD4bl and V3, other Env variable loops, such as V1V2, loop D, V4 and V5, may be explored for development of vaccine immunogens and therapeutics against HIV-1 infection.
